# Bilateral Ischemic Optic Neuropathy Developed under Interferon Therapy

**DOI:** 10.1155/2012/102739

**Published:** 2012-10-18

**Authors:** Fatih Selcukbiricik, Deniz Tural, Tuba Elif Senel, Ahmet Sarıca, Ozlem Soyluk, Suheyla Serdengecti

**Affiliations:** ^1^Division of Medical Oncology, Department of Internal Medicine, Cerrahpasa Faculty of Medicine, Istanbul University, 34300 Istanbul, Turkey; ^2^Department of Ophthalmology, Cerrahpasa Faculty of Medicine, Istanbul University, 34300 Istanbul, Turkey; ^3^Department of Internal Medicine, Istanbul Faculty of Medicine, Istanbul University, 34300 Istanbul, Turkey

## Abstract

*Introduction*. Interferon is a glycoprotein produced by assigned cells of immune system. It has been used in many different diseases. Although flu-like syndrome, myalgia, rash, hypotension, thrombocytopenia and peripheral neuropathy due to interferon use are encountered frequently, ocular side effects are rare, generally mild and transient. *Case Report*. 47-year-old female patient, presented with a mass lesion in right renal pelvis. Right radical nephrectomy was applied and the histopathological examination was consistent with papillary renal cell carcinoma. Interferon alpha treatment was started subcutaneously at the dose of 5 MIU/3 times in a week. Four weeks after the interferon therapy, suddenly bilateral visual loss developed. We discussed the diagnosis, followup, and treatment of the patient who developed irreversible ischemic optic neuropathy and had no previous known primary systemic disease to cause this condition. *Conclusion*. We suggest that patients should be screened for risk factors causing optic ischemic neuropathy, before interferon therapy. Although there was no adequate information in the literature for the followup, patients should be monitorized before, 1 month after, and 2 months after the treatment. And if there is no complication, we suggest that they should be followed up at 3-month intervals.

## 1. Introduction

Interferon is a glycoprotein produced by assigned cells of immune system. It has been used in treatments of hepatitis B, hepatitis C, hairy cell leukemia, follicular lymphoma, condyloma accuminata, Kaposi's sarcoma related to AIDS, renal cell carcinoma, and essential thrombocytosis treatment [1, 2]. Many side effects related to interferon use but ocular side effects are rare. It is observed in less than 1% of patients treated by interferon. Generally they are mild and transient. Risk of retinopathy has been higher in patients with diabetes mellitus, hypertension, dyslipidemia, and coagulopathy [3]. The treatment is composed of hydration, prevention of hypotension, and discontinuation of interferon treatment. Pulse steroid application is another option in the treatment. Prognosis of ischemic optic neuropathy related to interferon alpha is variable; some patients require discontinuation of interferon therapy but still do not improve although they have received relevant therapy.

## 2. Case

47-years-old female patient with Caucasian ethnicity applied with the complaint of abdominal pain for 2 months. A mass of 7 × 5.5 × 4 cm diameter was observed in the right renal-pelvis localization in computerized tomography examination. Patient had right radical nephrectomy, and histopathological examination was consistent with papillary renal cell carcinoma. In the positron emission tomography examination, lymphadenomegalies, which had intense hypermetabolic involvement, and malign (metastatic) features were detected in the nephrectomy location, abdominal lymphatics at the right side, bilateral mediastinal lymphatic locations, and left supra and retroclavicular fossae. Therefore, interferon alpha treatment was started subcutaneously at the dose of 5 MIU/3 times in a week with the preliminary diagnosis of metastatic renal cell carcinoma. 

Patient applied to the physician with suddenly developed bilateral visual loss 4 weeks after the interferon therapy. ESH 35 mm/h, Hgb 13.1 mg/dl, PT 13 sec, aPTT 30 sec, Protein S 80, Protein C 90, antithrombin 3, anti-phospholipid antibody (-), RF(-), ANA(-), p-ANCA(-), anti-dsDNA(-), CRP 3.2 mg/dl, and C3, C4 levels were within normal limits. Carotid doppler ultrasonography did not reveal any obstructions. Cranial MR and echocardiography revealed no pathological finding.

**Figure 1:**
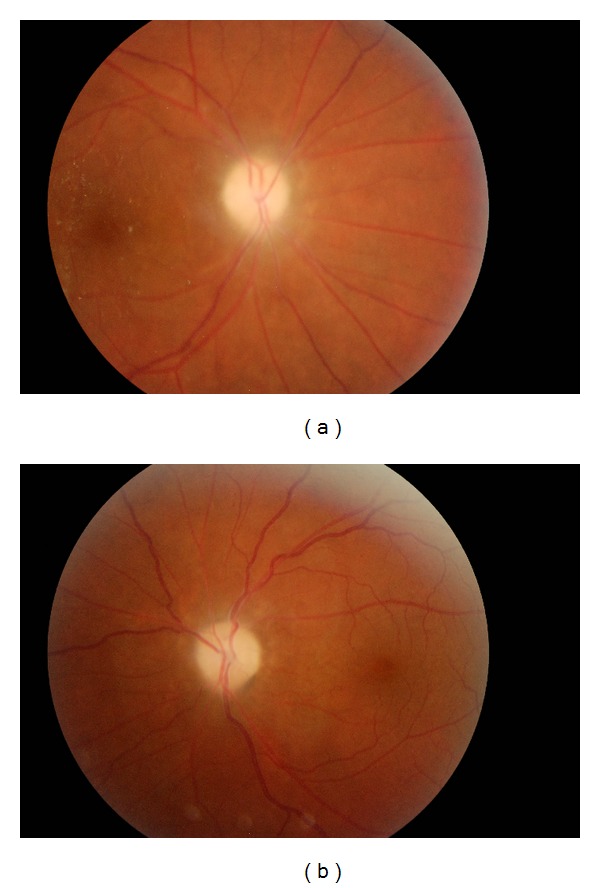
Bilateral ischemic optic neuropathy. The marked edema in the optic disc has a hemorragie near the papilla. Evanescence of optic border.

Ejection fraction was 55% in the echocardiography. The visual loss was decided to be due to interferon therapy, so interferon therapy was stopped and patient was consulted with the ophthalmology clinic. The patient had no previous visual problem, and the ophthalmological examination revealed visual acuity of 4/10 in the right and 2/10 in the left eyes with bilateral optic disc edema. Performed fluorescent angiography was consistent with bilateral ischemic optic neuropathy (Figures 1(a) and 1(b)). Patient received 1 gram methylprednisolone and intravitreal triestolon treatment for 5 days. In the ophthalmological examination, which was performed approximately 3 months after the ischemic optic neuropathy diagnosis, visual acuity was distinct decline in the right, and in the left eyes; and the condition was interpreted as irreversible.

## 3. Discussion

Interferon is a glycoprotein produced by assigned cells of immune system and plays a role in specific gene expression by activating intracellular signal pathway through membrane receptor binding, so it has antiviral, antiproliferative, and immunomodulatory activities. 

Frequently encountered side effects due to interferon use are fever, hypothermia, flu-like symptoms, myalgia, loss of appetite, fatigue, peripheral neuropathy, thrombocytopenia, nausea, vomiting, hypotension, tachycardia, thyroid function test disorders, depression, and leukopenia [2, 4]. Although ocular side effects are rare [5], frequently reported side effects in the ophthalmological literature are blurred vision, irritable conjunctivitis, and pain due to interferon secretion from tear gland [1]. Retinal ischemic changes are observed in less than 1% of patients who receive interferon therapy, and they are encountered in 2-week to 3 month time intervals after the initiation of interferon therapy. They may be spontaneously improved partially during the drug intake or after the discontinuation. Fluorescent angiography reveals ischemic changes. In the funduscopic examination, spectrum may vary between cotton-wool appearance to vascular occlusion, but central vision is generally not impaired [6].

There are many patients who developed asymptomatic or reversible retinopathy due to interferon in the literature. Atypical, severe, and rare ocular complications have also been reported. Oculomotor nerve paralysis, optic disc edema, subconjunctival vitreal hemorrhage, renal vein occlusion, and macular edema are present among them. Diabetes mellitus, hypertension, anemia, thrombocytopenia, and increased triglyceride levels are risk factors for retinopathy related to interferon use [7]. After excluding other etiologies for retinopathy, like vasculitis, demyelinization, infection, connective tissue diseases, and emboli, ischemic optic neuropathy due to interferon use was diagnosed depending on the symptoms and signs in the patient [8, 9].

Interferon induced ischemic optic neuropathy is believed to have a multifactorial pathophysiology depending on some ischemic factors. Suspected mechanisms for ischemia are interferon-induced lymphocyte and vascular adhesion molecule activation, increased immune complex circulation in the region as a result, and accumulations of these in optic artery or small retinal arteries. Moreover, interferon elicits increases in some interleukins and MHC2 proteins by its immunomodulation feature [10]. One of the other ischemic pathophysiological mechanisms that interferon is believed to induce optic nerve ischemia is by causing systemic hypotension and fluctuations in blood pressure. Sugano et al. [11] defined in their study conducted on patients with retinopathy and under interferon therapy that active c5 [6] protein levels, which led to intravascular thrombocyte aggregation, retinal capillary infarct by damaging retinal blood circulation, cotton-wool appearance, and hemorrhages at the fundus, were abnormally high [12, 13].

Treatment approach to patients with interferon-related ischemic retinopathy is composed of hydration treatment, prevention of hypotension, and discontinuation of interferon therapy. Pulse steroid application is also an option for the treatment. Prognosis of ischemic optic neuropathy due to interferon alpha is variable. While visual loss is improved in some patients, although interferon is discontinued and treatment is started, no improvement can be observed. Visual loss has been permanent in our patient.

In conclusion, we suggest that patients who would receive interferon therapy should be screened for risk factor for optic ischemic neuropathy before the treatment; and although there was no adequate information in the literature for the followup, patients should be monitored before, 1 month after, and 2 months after the treatment. And if there was no complication, then we suggest that patients should be followed up at 3-month intervals.

## 4. Summary

Interferon (IFN) is a glycoprotein produced by assigned cells of immune system and it has antiviral, antiproliferative, and immunomodulatory effects. Although flu-like syndrome, myalgia, rash, hypotension, thrombocytopenia, and peripheral neuropathy due to interferon use are encountered frequently, ocular side effects are rare, generally mild, and transient. In our case report, we discussed the diagnosis, followup and treatment of a 47-year-old patient, who received IFN therapy with the diagnosis of metastatic renal cell carcinoma, developed irreversible ischemic optic neuropathy, and had no actual primary systemic disease to cause the condition with the literature.
